# Extraction Techniques, Biological Activities and Health Benefits of Marine Algae *Enteromorpha prolifera* Polysaccharide

**DOI:** 10.3389/fnut.2021.747928

**Published:** 2021-10-07

**Authors:** Teketay Wassie, Kaimin Niu, Chunyan Xie, Haihua Wang, Wu Xin

**Affiliations:** ^1^Key Laboratory of Agro-Ecological Processes in Subtropical Region, National Engineering Laboratory for Pollution Control and Waste Utilization in Livestock and Poultry Production, Hunan Provincial Engineering Research Center for Healthy Livestock and Poultry Production, Institute of Subtropical Agriculture, Chinese Academy of Sciences, Changsha, China; ^2^Institute of Biological Resources, Jiangxi Academy of Sciences, Nanchang, China; ^3^Tianjin Institute of Industrial Biotechnology, Chinese Academy of Sciences, Tianjin, China; ^4^Qingdao Seawin Biotech Group Co., Ltd., Qingdao, China

**Keywords:** biological activity, enteromorpha prolifera, extraction technique, health benefit, microbiota, sulfated polysaccharide

## Abstract

There is increasing interest in the use of marine algae as functional food additives for improving human health. *Enteromorpha (Ulva) prolifera* (*E. prolifera*) is a seaweed green alga (*Chlorophyta)* that contains many bioactive compounds, of which polysaccharide is the main component. With the advancement of technology in the methods of extraction and analysis, recent studies in *in vitro* and animals model showed that polysaccharides derived from *E. prolifera* exert various biological activities, such as gut microbiota modulation, immunomodulation, antioxidant, antidiabetic, antimicrobial, and hypolipidemic. Research evidence has shown that methods of extraction and molecular modification, such as degradation, carboxymethylation, and sulfonation could alter the biological activities of polysaccharides. Therefore, in this review, we discussed the different extraction techniques, structural-activity relationship, and health benefits of sulfated polysaccharides derived from *E. prolifera*, and suggested future research avenues. This review helps to advance the extraction techniques and promote the application of marine algae polysaccharides as functional food and therapeutic agent.

## Introduction

Algae are plants of marine benthoses, which can be classified as unicellular microalgae and macroalgae. Macroalgae are multicellular aquatic photosynthetic organisms that are included under Plantae and Chromista kingdoms (1). According to the nature of their pigments, macroalgae are divided into three major groups: green algae (*Chlorophyta*), red algae (*Rhodophyta*), and brown algae (*Phaeophyceae*) (2, 3). The growth and distribution of green algae (*Chlorophyta*) in the marine environment as a green tide have been reported worldwide (4–7). This macroalgal bloom causes a devastating effect on the marine ecosystem due to shading, biomass decomposition, and anoxia (8), in aquatic microbial ecosystem shift (9), and macrofauna inhibition (10). *Enteromorpha* as a genus name belonging to the phylum Chlorophyta, class Chlorophyceae, order Ulvales, is a seaweed green alga distributed worldwide (11). Under the genus *Enteromorpha*, there are different species of green algae such as *E. prolifera, E. intestinalis, E. linza, E. flexuosa* and *E. compressa*. Morphological and molecular analyses have revealed that *E. prolifera* is the dominant species of green tides in the Yellow Sea of China (12). Although *E. prolifera* tide put a threat to the marine ecosystem, it has been used as traditional medicine and functional food (13–17). Recently, the nutritional composition and safety of *E. prolifera* have been investigated, and has been found that *E. prolifera* contains essential nutrients such as carbohydrate (43–51%), protein (26–33%), fat (0.2–0.8%), total amino acid (20.26–23.32%), ash (13–14%) and iron (1.1–3.4mg/g) (18), of which carbohydrate is the most abundant component (19). Furthermore, health risk assessment studies showed that the level of major micropollutants such as heavy metals, pesticides, and polycyclic aromatic hydrocarbon in *E. prolifera* is below the limit to cause health hazards and thus can be regarded as safe for human consumption (18).

Polysaccharide is an essential biomacromolecule, which is formed from multiple monosaccharides linked by glycosidic bonds. Collective evidence has shown that polysaccharides are the main biologically active molecules of *E. prolifera* (11). These polysaccharides are found in the algae as a cell wall structural component. The chemical composition analysis using reverse-phase high-performance liquid chromatography (HPLC) and gas chromatography showed that *E. prolifera* contains sulfated polysaccharides mainly composed of rhamnose (Rha), glucose (Glc), galactose (Gal), and xylose that are linked by glycosidic bonds (20). Furthermore, it has been confirmed that *E. prolifera* polysaccharides exert various pharmacological activities, such as antioxidant, antidiabetic, antimicrobial, immunomodulatory, and hypolipidemic (21–23). The biological activity of polysaccharides depends on physicochemical and structural characteristics. Therefore, the molecular modification of these polysaccharides provides a way to improve their bioactivity. For example, enzymatic degradation and sulfonation of *E. prolifera* polysaccharides could enhance the antioxidant activity (20, 24).

Polysaccharides can be extracted from *E. prolifera* using different methods, including hot water, alkali, acid, enzyme-assisted, and microwave-assisted methods. It has been reported that the extraction methods and conditions could alter the composition, yield, and molecular weight of extracted polysaccharides (25).

*E. prolifera* polysaccharides have been increasingly investigated for their role in the functional food and pharmaceutical industry (26). For example, *Enteromorpha* species was used as an ingredient in the preparation of pakoda, a common traditional snack food in India (27). Hence, in this review, we summarized the extraction methods, structural-activity relationship, biological activities, and health benefits of *E. prolifera* polysaccharides and pointed out future research directions.

## Extraction Methods

**Figure 1:**
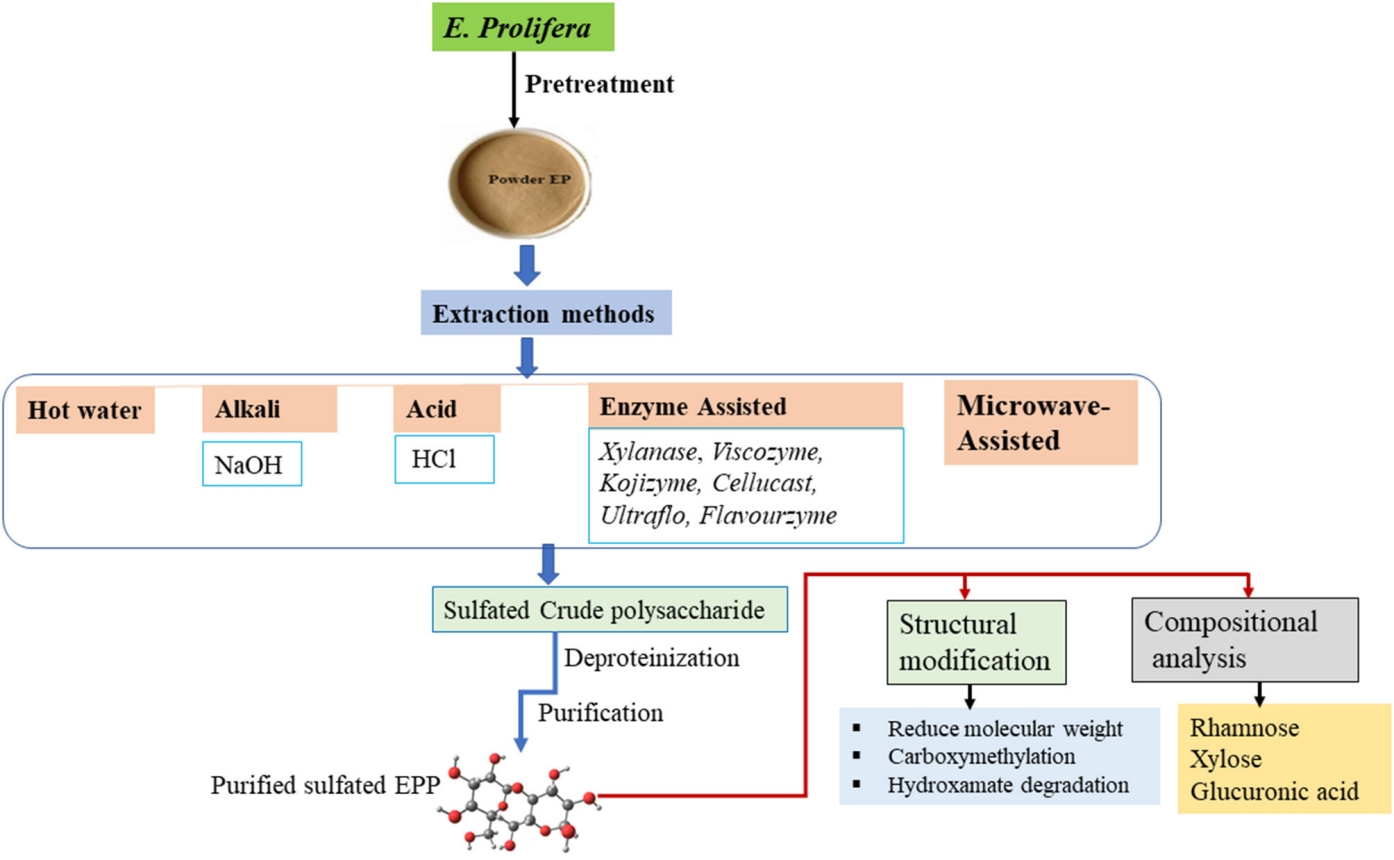
Schematic diagram of the extraction, purification, and modification of *Enteromorpha prolifera* polysaccharide (EPP).

Different methods have been used for the extraction and preparation of polysaccharides from *E. prolifera*, which have a significant influence on the yield, molecular weight, and composition. The types of extraction techniques currently in use are presented in Figure 1. Steps in the general procedure for the extraction of polysaccharides include washing for the removal of impurities, disruption of cellular component, extraction of polysaccharides into external solvent medium, and finally purification (28).

The effect of extraction methods on the composition and biological activity of *E. prolifera* polysaccharides was recently examined. A study by Chi et al. (25) showed that the acid extraction method produced a better yield and higher molecular weight *E. prolifera* polysaccharide with intense iron chelating capacity compared with their respective water and alkali methods. Apart from extraction techniques, extraction conditions, such as time, temperature, pH, the ratio of biomass amount to solvent, and the type of elution solvent could affect the chemical composition and function of polysaccharides (19, 29). For example, Yuan and his colleagues evaluated the effects of extraction temperature on the monosaccharide composition of *E. prolifera* polysaccharides (30). The authors found that rhamnose, galacturonic acid, and glucose were the main monosaccharides of *E. prolifera* extracted at 90°C. However, glucose became the only major monosaccharide when the extraction temperature increased to 150°C (30). In addition, the concentration of elution solvent affects the monosaccharide composition and content. Recently, Zhao et al. (26) extracted crude polysaccharide from *E. prolifera* using the alkali method and fractionated it by stepwise elution with 0 (AP-1), 0.3 (AP-2), 0.5 (AP-3), 0.7 (AP-4) M NaCl solution through an anion-exchange column. The monosaccharide analysis indicated that PAP-1 and PAP-2 mainly consist of galacturonic acid, while PAP-3 and PAP-4 mainly contained rhamnose. Furthermore, rhamnose and xylose were not detected in polysaccharides eluted with 0.3 M NaCl. Similarly, Cho et al. (31) extracted *E. prolifera* polysaccharide with hot water method and eluted with distilled water (F1), 0.5 mol/L NaCl (F2), and 1.0 mol/L NaCl (F3). The authors found that rhamnose was the major neutral sugar of the F1 (65.7%) and F2 (57.1%) fractions with considerable amounts of glucose (31.9%) and (39.1%), respectively. On the other hand, % of rhamnose was highest in F3 (87.6%) with small percentages of xylose and glucose. Taken together, the above studies show that polysaccharides with diverse monosaccharide compositions and content can be obtained by different extraction methods, conditions, and elution solvent concentrations.

The extraction parameters not only affect the composition of the polysaccharide, but also its biological activity. For example, the elution solvent's effect on polysaccharide's antioxidant activity was explored after hot water extraction and DEAE-52 chromatography purification. The purified polysaccharide was eluted with different concentrations (0, 0.1, and 0.3 mol/L) of NaCl, and three fractions of purified polysaccharides were obtained. The physiochemical and free radical scavenging analysis showed that these three fractions possessed different molecular weights and demonstrated various degrees of antioxidant activity, suggesting that the elution solvent influences the structure and biological activity of a polysaccharide (32). Table 1 shows *E. prolifera* polysaccharide extraction methods and parameters.

**Table 1:** Summary of the extraction methods and conditions for the isolation of polysaccharides from *Enteromorpha prolifera*.

**Name**	**Method of extraction**	**Time**	**Temperature**	**Algae/water ratio (g/mL)**	**pH**	**Solvent**	**Yield**	**Sulfate content (%)**	**Ref**
Crude polysaccharide	Hot water	2 h	65°C	1:20	-	Water	25.1%	15.1	(31)
HWP	Hot water	2 h	100°C	1:30	-	Water	21.3%	16	(25)
EP	Hot water	3 h	100°C	1:30.03		Water	10%	15.53	(20)
AKP	Alkali	3 h	60°C	1:40		Water	10.7%	19.1	(25)
AP	Alkali	3 h	Room temperature	1:10		Water	-		(26)
ACP	Acid	1 h	80°C	1:20		Water	24.7%	16.2	(25)
EP	Acid	1	Room temperature	1:30		Water	20.1%	18.99	(33)
EAP	Enzyme assisted using cellulase	1.5 h	50°C	1:30	5.0	Water	21.4%	16.7	(25)
EAE	Enzyme assisted using cellulase	8 h	50°C	1:50	4.8	Citrate buffer	36%		(34)
Crude	Microwave- assisted	15 min	120°C	1:20		0.01 HCl	36.38%	6.46	(30)

*HWP, Hot-water extraction; EAP, Enzyme-assisted extracted polysaccharide; ACP, Acid extracted polysaccharide; EAE, Enzyme-assisted extraction; EP, E. prolifera polysaccharide; AKP, Alkali extracted polysaccharide; AP, Crude polysaccharide*.

### Hot-Water Extraction

This method used hot water to break the cell wall component of the algae to release the intracellular molecules into the solvent. According to Cho et al. (31), the collected algae were washed with distilled water and dried at 60°C. The dried samples were then minced to get homogenate powder and filtered using a sieve (<0.5 mm). Next, the powder was soaked in hot water (100°C, alga density 33.3 g/L) for 3 h (20) or (1:20 algae to water ratio, at 65°C for 2 h) with constant mechanical stirring (31). According to Chi et al. (25), the extraction conditions were: ratio of algae to water, 1:30; extraction temperature, 100°C; and extraction time, 2 h. The solution was cooled, centrifuged at 18,500 × g for 10 min, and the supernatant was collected and precipitated with 95% ethanol (1:4, v/v) for 24 h at 4°C. The precipitate was dried at room temperature (24 h) overnight to obtain crude polysaccharides. The crude polysaccharide was deproteinized by sevage method [chloroform: butyl alcohol, 4:1 (v/v)] (35). Depending on the purpose, different fractions of polysaccharides could be obtained by various treatments of crude polysaccharides. For example, Cho et al. (31) dissolved 100 mg crude polysaccharide with 10 mL distilled water and fractionated using ion-exchange chromatography on a DEAE Sepharose fast flow column. Then, they washed the column and eluted the polysaccharide with distilled water (F1), 0.5 (F2), and 1.0 M (F3) NaCl. This method has been reported to yield 10% (20), 21.3% (25), and 25.1% (31) crude polysaccharides. The inconsistency in yield may be attributed to the difference in extraction conditions. However, fractionating and elution solvent concentrations can also affect the extraction yield of polysaccharides. For example, the yields of polysaccharides eluted with distilled water, 0.5 and 1.0 M NaCl were 7.3, 32, and 46.7%, respectively (31). The average molecular weight of crude polysaccharides obtained in this method was 966 kDa (25).

### Alkali Extraction

Alkali extraction techniques used NaOH to isolate polysaccharides from *E. prolifera* (36). The principle of this method is that the hydroxyl ions (OH^−^) of the base interfere with hydrogen linkages in the polysaccharides to release it into the solvent. Briefly, the algae were dried and subjected to extraction with 95% ethanol at 60°C for 2 h and distilled water at 90°C for 2 h. According to Zhao et al. (37), the algae were further washed, dried (overnight at 60°C), and treated with NaOH solution (0.30 mol/L) at room temperature for 3 h with 1:10 (w/v) algae to solvent ratio. However, the extraction conditions reported by Chi et al. (25) were: 0.5 M NaOH solution; 1:40 algae to solvent ratio; temperature, 60°C and time, 3 h. The residue was then filtered, and the alkali was neutralized with 0.1 mol HCl. The crude extract was then centrifuged (4,800 rpm, 10 min), the supernatant was collected and precipitated with 95% ethanol (1:4, v/v) for 24 h at 4°C. Following centrifugation, the precipitated solution was further washed with 95% ethanol and freeze-dried (−50°C for 24h). The extracted crude polysaccharide was deproteinized by chloroform: butyl alcohol (4:1, v/v) (38). Next, the polysaccharide was fractioned in DEAE-52 cellulose and eluted with distilled water (500 mL) and 0.3, 0.5, 0.7 M NaCl at a flow rate of 1.0 mL/min (37). Finally, the fractionated polysaccharides were purified through the Sephadex G-100 column (2.5 × 60 cm) and then concentrated, dialyzed, and lyophilized. According to Zhao et al. (37), average molecular weights of polysaccharides extracted with distilled water (500 mL), 0.3, 0.5, 0.7 M NaCl were 34.4, 64.2, 120, and 48.2 kDa, respectively. In addition, *E. prolifera* extracted with this method was found to yield 10.7% crude polysaccharide with an average molecular weight of 47.7 kDa (25). These results demonstrated alkali method is less efficient and produced a low yield (10.7%) compared with acid (24.7%) and enzyme-assisted extraction methods (36%) (25).

### Acid Extraction

The principle of this method is that the acid (HCl) penetrates the algae cell component and then the H^+^ of the acid interferes with hydrogen linkages in polysaccharide to release it into the solvent. Different researchers isolated polysaccharides using this method in different time-temperature combinations. Briefly, Liu et al. (33) reported that polysaccharides could be extracted from *E. prolifera* using 0.1 N HCl at room temperature for 4 h in 1:30 (w/v) algae: water ratio. Recently, Chi and his colleagues isolated polysaccharides from *E. prolifera* with 0.1 M HCl solution at 80°C for 1h and sample to solvent ratio of 1:20 (w/v) (25). Following filtration, the collected crude polysaccharide was treated with 6 N NaOH to neutralize the acid. After centrifugation (4,800 rpm, 10 min), the supernatant was recovered and precipitated with absolute ethanol. The crude polysaccharide was then deproteinized by treating with chloroform: butyl alcohol (4:1, v/v) (35). Next, polysaccharide was purified using anion exchange chromatography on a DEA Bio-Gel Agarose FF gel and eluted with 1 M NaCl. Then, the polysaccharide was precipitated by 95% ethanol (1:4, v/v) for 24 h at 4°C, dialyzed with distilled water, and freeze-dried (−50°C for 24 h) (25). The acid extraction method was found to produce of 24.7% yield with an average molecular weight of 41.1 kDa (25). Liu et al. (33) reported that the extraction yield and molecular weight of the polysaccharide obtained by this method were 36.0% and 17.3 kDa, respectively. As compared with hot water and alkali extraction methods, the acid extraction method was found to produce better yield, high molecular weight *E. prolifera* polysaccharides with iron chelating capacity (25).

### Enzyme-Assisted Extraction

The principle of this method is that the enzymes degrade the algae's cell wall structure, thereby releasing the intracellular molecules to the solvent (39, 40). Currently, various enzymes have been used for the extraction of polysaccharides from *Enteromorpha prolifera*, including *xylanase, viscozyme, kojizyme, cellucast, ultraflo, flavourzyme termamyl, protamex, cellulase*, and *neutrase* (41). However, the selection of appropriate hydrolytic enzymes significantly affects the extraction efficiency. Furthermore, enzymes work at a specific pH, and the time-temperature combination also influences the rate of enzyme reaction. The optimum pH and time-temperature requirements of different enzymes were summarized previously (40).

In an enzyme-assisted extraction method, the *E. prolifera* samples were washed, 2 g sample was dispersed in 50 mL distilled water and incubated in an agitated water bath for 10 min, dried in an oven at 60°C, and milled to <1 mm particle size. Appropriate enzymes were then selected, and the time-temperature combination was adjusted. According to Michalak et al. (34), for enzyme-assisted extraction using cellulase (*Trichoderma reesei ATCC 26921*; Sigma-Aldrich Chemie GmBh, Schnelldorf, Germany), the recommended optimal extraction conditions were; the algae: enzyme: water ratio was 1 g:25 μL:50 mL, pH 4.6 and 8 h extraction time. The enzyme was prepared by dissolving in sterile deionized (DI) water in the presence of 0.15% polyhexamethylene biguanide at 5 mg/mL concentration. In another study by Chi et al. (25) for *E. prolifera* extraction using cellulase (Novozymes Co., Ltd., Copenhagen, Denmark), the enzyme amount was 2% (w/v), the ratio of algae to water was 1:30 (w/v), and the extraction was performed at 50°C for 1.5 h at pH 5.0. The enzyme reaction was then inactivated by heating the reaction at 90–100°C for 10 min, and then immediately cooled on an ice bath. Finally, the solutions were centrifuged (4,800 rpm, 10 min) and filtered to collect the crude polysaccharides (24). The crude polysaccharides could be further degraded with hydrogen peroxide and ascorbic acid to produce polysaccharides of different molecular weights (24, 36). The yield obtained using enzyme assisted method was found to be 36% (34). Chi et al. (25) reported the yield and average molecular weight of *E. prolifera* polysaccharide extracted by this method to be 21.45% and 1,327.5 kDa, respectively. Compared with the above-mentioned methods, this method is efficient in terms of extracting high molecular weight polysaccharides because of less off-target degradation. However, consideration should be given to select appropriate enzymes and optimize influencing factors to improve the extraction efficiency and the quality of extracted molecules.

### Microwave-Assisted Extraction

This method used microwave energy to heat solvents in contact with a sample to partition analytes from the sample matrix into the solvent (30). Briefly, the algae sample was prepared by washing, air drying, and trituration/grinding. Then, the algae powder was suspended in 0.01 HCl solution (1:20, algae: solvent ratio), thoroughly mixed, and placed into a 200 mL microwave tube. The solution was then exposed to irradiation (2.45 GHz) using a UWave-2000 reactor at 120°C for 15 min. The solution was agitated at 300 rpm using a magnetic stirring bar during irradiation. Following irradiation, the residue was separated by centrifugation (6,000 × g for 20 min) and dried at 80°C. The supernatant was collected and neutralized by 1 M NaOH. The solution was then precipitated with 95% ethanol (1:2 v/v), washed, and lyophilized to obtain crude polysaccharides. The yield and average molecular weight of polysaccharides obtained in this method were 36.38% and 156 kDa, respectively (30). However, extraction temperature and acid concentration significantly influenced the yield and molecular weight of extracted material. For example, at 150°C and 0.1 M HCl extraction conditions, the yield and average molecular weight of the polysaccharide were 6.09% and 10.5 kDa, respectively (30). Therefore, the extraction condition should be optimized to get maximum yield. Generally, if the extraction conditions are optimized, this method is more efficient than the other methods in terms of yield.

## Structure-Activity-Relationship of Native and Modified *E. Prolifera* Polysaccharides

The polysaccharide is an essential organic compound for life, composed of multiple monosaccharides of the same or different types. The type, sequence, average molecular weight, and combination of monosaccharides determine the physicochemical and structural characteristics of polysaccharides, by which the structure influences its function. It has been reported that seaweed polysaccharides are mainly sulfated polysaccharides (42). *E. prolifera* contains both water-insoluble and water-soluble carbohydrates as a primary component. The water-insoluble portion of *E. prolifera* is cellulose and hemicellulose, whereas the water-soluble part mainly consists of sulfated polysaccharides and a small amount of starch (43). The chemical composition analysis showed that polysaccharide is the main chemical component of *E. prolifera* that accounts for more than 50% of the dry weight (11).

**Figure 2:**
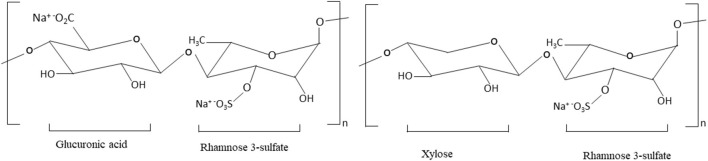
The structure of *E. prolifera* polysaccharide. This polysaccharide is composed of monosaccharides: rhamnose, glucuronic acid, and xylose that are linked by β (1–4) glycosidic bonds. The C-3 position of rhamnose contains a negatively charged sulfate group.

The backbone of *Enteromorpha* (*ulvan*) polysaccharides is composed of α- and β-(1, 4)- linked monosaccharides (rhamnose, xylose, glucuronic acid, and iduronic acid) (44, 45). The structure of green seaweed *Enteromorpha prolifera* is not yet well-reviewed. Yu et al. (11) reported that the *E. prolifera* polysaccharide backbone is composed of D-GlcUAp-α-(1 → 4)-3-sulfate-l-Rha p- β-(1 → 4)-d-Xyl p-β-(1 → 4)-3-sulfate-l-Rha p units. The structure of *E. prolifera* polysaccharide is depicted in Figure 2 and the structural characteristics of different polysaccharides isolated from *E. prolifera* are presented in Table 2. In *E. prolifera* polysaccharide, the sulfate group is attached at the C-3 position of rhamnose (11), which is similar to the polysaccharide from *E. intestinalis* (38), but different from polysaccharide from *E. clathrate*, where the sulfate group is attached at the C-3 position of arabinose (49) *and E. compressa* polysaccharide, where the sulfate group is attached at the C-3 of rhamnose and the C-2 of xylose (50, 51). Furthermore, some *ulvans* polysaccharides such as *E. compressa* polysaccharides are branched containing glucuronic acid (50, 52), whereas *E. prolifera* polysaccharide has no branch.

**Table 2:** The structure and monosaccharide composition of different polysaccharides isolated from *Enteromorpha prolifera*.

**Name**	**Assay method**	**M.W**	**Monomer units**	**Biological activity**	**Reference**
PEP	HPLC	147kDa	Rha, Glu, Gal, Xyl and Ara	Anti-oxidant and moisture absorption/retention capacities	(20)
			1.48:1:0.13:0.3:0.06		
SPEP	HPLC	176.3kDa	Rha, Glu, Gal, Xyl, Ara		
			1.49:1:0.16:0.85:0.07		
LEP		44.8kDa	Rha, Glu, Gal, Xy, Ara		
			1.65:1:0.09:0.57:0.17		
SLEP		59.9 kDa	Rha, Glu, Gal, Xyl and Ara		
			1.09:1:0.06:0.1:0.11		
EPF2	Gas chromatography	103.51 kDa	Rha, Xyl, Man, Gal, and Glu	Hypolipidemic and antioxidant	(46)
			3.64:1.08:0.21:0.75:0.27		
EPP-1	HPLC	4.28 kDa	Man, Rha, GlcUA, GalUA, Glu, Gal	Antioxidant and anti-aging	(47).
			0.61: 12.53: 30.59: 3.26: 1.73: 21.69		
HWP	HPLC	966.0 kDa	Rha, GlcUA, Glu, Xyl	Metal-ion chelating capacity	(25)
			1: 0.31: 1.29: 0.49		
EAP		1327.5 kDa	Rha, GlcUA, Glu, Xyl		
			1:0.25: 0.85: 0.40		
ACP		41.1 kDa	Rha, GlcUA, Glu, Xyl		
			1:0.37: 1.16: 0.23		
AKP		47.7 kDa	Rha, GlcUA, Glu, Xyl		
			1:0.37:0.23:0.40		
SUE	HPLC	1340 kDa	Rha, Glu, GlcUA, Xyl.	Anti-anemia	(48)
			57.9%:12.1%:16.3%:13.7%		
Crude	HPLC	1218 x 10^3^g/mol	Rha, Xyl, Glu	Nitric oxide production	(31)
			70.20 ± .6%: 3.50 ± .3 %:26.30 ± .30		
F1		826 x 10^3^g/mol	Rha, Xyl, Glu		
			65.73 ± .2%: 2.40 ± .1%: 31.93 ± .10 %		
F2		1,281 x 10^3^g/mol	Rha, Xyl, Glu		
			57.11 ± .3%: 3.80 ± .3%: 39.11 ± .0%		
F3		786 x 10^3^g/mol	Rha, Xyl, Glu		
			87.60 ± .6%: 8.80 ± .5%:3.60 ± .1		
DEP1	HPLC	446.5kDa	Rha, Man, Glu, Gal, Xyl, Fuc	Antioxidant	(43)
			1:0.047: 0.89: 0.074:0.32:0.0145		
DEP2		247kDa	Rha, Man, Glu, Gal, Xyl, Fuc		
			1:0.044: 0.91: 0.074: 0.33 0.0171		
DEP3		76.1kDa	Rha, Man, Glu, Gal, Xyl, Fuc		
			1:0.045: 0.91: 0.077: 0.31: 0.0143		
PE	Gas chromatography	1400 kDa	Rha, Glu, Xyl, Gal, Man	Antioxidant	(24)
			67.8:18.6: 7. 7: 4.0:1.4		
DPE		44 kDa	Rha, Glu, Xyl, Gal, Man		
			56.9: 31.8: 6.4: 2.5:2.5		
EP1	HPLC	8 kDa	Rha, GlcUA, Gal, Xyl	Immunomodulatory	(33)
			1: 0.29: 0.07: 0.27		
EP2		4 kDa	Rha, GlcUA, Gal, Xyl		
			1: 0.2: 0.01:0.56		

*M.W, Molecular weight; HPLC, High-performance liquid chromatography; Rha, Rhamnose; Glu, Glucose; Gal, Galactose; Man, Mannose; Fuc, Fructose; Xyl: Xylose; Ara: Arabinose; GlcUA, Glucuronic acid; GalUA, Galacturonic acid; PEP, Enteromorpha prolifera polysaccharide; SPEP, sulfated E. prolifera polysaccharide; LEP, Low molecular weight polysaccharide; SLEP, Sulfated low molecular weight polysaccharide; EPF, E. prolifera polysaccharide fraction; EPP-1 E. prolifera polysaccharide1; PE, E. prolifera polysaccharide; HWP, Hot-water extracted polysaccharide; EAP, Enzyme-assisted extracted polysaccharide; ACP, Acid extracted polysaccharide (ACP); AKP, Alkali extracted polysaccharide; SUE, Sulfate Ulva polysaccharide; F, Polysaccharide fraction; DEP, E. prolifera with different molecular weight; UPR, Ulva prolifera residue; DPE, Degraded polysaccharide; EP, Enteromorpha prolifera polysaccharide*.

The biological activities of polysaccharides are determined by the structure, such as the sulfate group, acetyl group, monosaccharide composition, and molecular weight (53). Therefore, any modification of polysaccharide structure and composition results in changes in its biological activities. Different methods of *E. prolifera* polysaccharides modification, such as carboxymethylation, hydroxamate modification, enzymatic hydrolysis, and sulfonation have been reported and the structure-bioactivity relationships have been studied.

Recently, the effect of degradation and carboxymethylation of polysaccharides isolated from *E. prolifera* on the antioxidant activities was evaluated by Shi et al. (24). The polysaccharides were extracted using the hot water method, degraded by hydrogen peroxide/ascorbic acid, and further carboxymethylated. The results indicated that both degradation (reducing molecular weight) and carboxymethylation could enhance the free radical scavenging ability of polysaccharides, as reflected by higher antioxidant activities of degraded carboxymethylated polysaccharides compared with degraded only and undegraded polysaccharides (24). It has been reported that hydroxamate modification could further improve the biological activity of *E. prolifera* polysaccharides. For this purpose, Shao et al. (22) modified carboxymethylated *E. prolifera* polysaccharide by hydroxylamine hydrochloride and examined its antioxidant activity. The authors demonstrated that the free radical scavenging and total antioxidant activities of the hydroxamate-modified polysaccharide were higher than the carboxymethylated modified one.

In another study, the effects of molecular weight on free radical scavenging and chelating activities of polysaccharides were examined. For this purpose, two polysaccharides from *E. prolifera* with different molecular weights but similar sulfate groups and monosaccharide composition were extracted, and their antioxidant ability was evaluated. The results showed that polysaccharides with lower molecular weight displayed an intense hydroxyl radical scavenging activity and chelating effects than higher molecular weight (43). It has also been reported that the acetyl group or sulfate group modification could improve the antioxidant activities of polysaccharides (54). Hot water extracted *E. prolifera* polysaccharide was hydrolyzed by *P. pabuli* enzyme, sulfonated with chlorosulfonic acid/pyridine, and then tested for biological activity (20). The authors demonstrated that enzymatic degradation and sulfate group modification improved free radicals scavenging activities *in vitro*, and enhanced moisture absorption capacities of the polysaccharides. In addition, Cui et al. (55) reported that enzymatic hydrolysis of sulfated *E. prolifera* polysaccharides by *Alteromonas sp. A321* could enhance anticoagulant activity *in vitro*. The above studies suggest that structural modification of polysaccharides could enhance their biological activities.

## Biological Activities and Health Benefits

**Figure 3:**
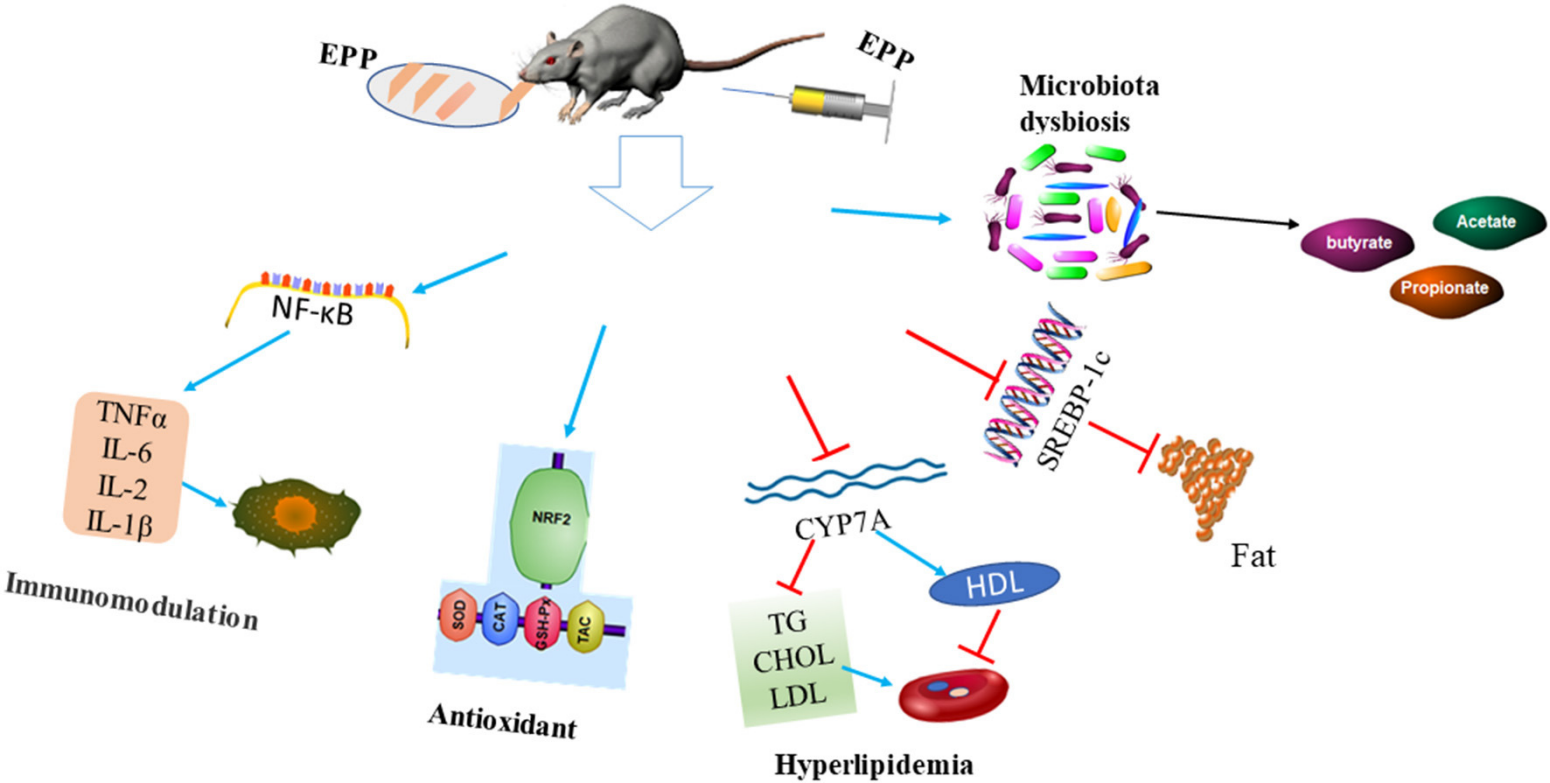
Biological activities and health benefits of *E. prolifera* polysaccharides. The injection or dietary supplementation of *E. prolifera* polysaccharides modulate gut microbiota and stimulate short-chain fatty acid production. In addition, it stimulates antioxidant activities, alleviates inflammation, reduced serum TG, CHOL, and LDL levels, and reduces fat accumulation *via* a different mechanism. EPP: *E. prolifera* polysaccharide; NF-κB: Nuclear factor kappa-light-chain-enhancer of activated B cells; IL-2: Interleukin 2; IL-6: Interleukin; IL-10: Interleukin; NRF2: Nuclear factor erythroid 2-related factor 2; SOD: Superoxide dismutase; CAT: Catalase; GSH-Px: Glutathione peroxidase; TAC: Total antioxidant capacity; TG: Triglyceride; CHOL: Cholesterol; LDL: Low-density lipoprotein; HDL: High-density lipoprotein; CYP7A: Cholesterol 7 alpha-hydroxylase; SREBP1c: Sterol regulatory element-binding protein-1c.

Several lines of evidence have shown that apart from being used as a food, *E. prolifera* polysaccharides could also perform different bioactive functions, such as immunomodulatory, antidiabetic, antioxidant, hypolipidemic, antimicrobial, and gut microbiota modulation (32, 38). The biological activities and health benefits of *E. prolifera* polysaccharides are presented in Figure 3.

### Immunomodulatory Activity

The role of sulfated *E. prolifera* polysaccharide as an immune stimulator has been recognized previously by different studies. Sulfated polysaccharides were extracted from *E. prolifera* using hot water and their immunomodulatory activity was explored on RAW 264.7 macrophages cells and in mice. It has been demonstrated that RAW 264.7 macrophages cell that received sulfated polysaccharides exhibited upregulated macrophage proliferation, as well as increasing nitric oxide secretion (21). The *in vivo* results further confirmed that the administration of sulfated *E. prolifera* polysaccharide to mice could enhance cell proliferation besides increasing interferon-alpha (IFN-α) and interleukin-2 (IL-2) secretions (21). Similarly, Liu et. (33) demonstrated the immunomodulatory effect of *E. prolifera* polysaccharide on RAW 264.7 macrophages and cyclophosphamide (CYP)-induced immunosuppression mouse models, found that *E. prolifera* polysaccharide could promote the secretion of interleukin-1beta (IL-1β), interleukin (IL-6), and tumor necrosis factor-alpha (TNF-α) *via* activation of *TLR4/MAPK/NF-*κ*B* signaling pathway in RAW 264.7 macrophages cells and mice.

In another study to prove the immunomodulatory effects of *E. prolifera* polysaccharide, the sulfated polysaccharide was extracted by a hot water method and administered to mice. The result showed that *E. prolifera* polysaccharide treatment could stimulate splenocyte proliferation (13, 21). Moreover, administration of mice with low molecular weight sulfated *E. prolifera* polysaccharide was found to increase splenocytes interferon-γ (INF-γ) and interleukin-2 (IL-2) production (56).

Further immune-related enzyme analysis showed that the treatment of mice with *E. prolifera* polysaccharide could enhance Nuclear factor-kappa B (NF-κB) expression and alkaline phosphatase (AKP), superoxide dismutase (SOD), and lactate dehydrogenase (LDH) production in a dose-independent manner, suggesting that the immunomodulatory effect of *E. prolifera* polysaccharide might be closely related to the upregulation of NF-κB transcription factor (13). Overall, this study confirmed the effect of *E. prolifera* polysaccharides on the humoral and cell-mediated immune response in mice.

In addition to studies *in vitro* and mice, it has also been reported that the administration of *E. prolifera* polysaccharides could enhance the non-specific immunity of sea cucumbers, thereby protecting sea cucumbers from splenic *vibrio* infection (57). Taken together, studies have confirmed that polysaccharide isolated from *E. prolifera* is a potential immunomodulator that can induce both humoral and cellular immunity.

On the other hand, a study showed that *E. prolifera* polysaccharide treatment protects human cardiac microvascular endothelial cells from oxygen-glucose deprivation-induced viability loss, proliferation inhibition, apoptosis, inflammatory cytokine expression *via* up-regulation of HIF-1α, and inactivation of the NF-κB pathway (58). Overall, these studies suggest that *E. prolifera* polysaccharides might play both immunomodulatory and anti-inflammatory roles depending on the physiological condition of experimental animals or cells.

### Hypolipidemic Activity

Abnormally high levels of lipids or fats can cause hyperlipidemia, a chronic disease that elevates the risk of heart disease and stroke (59). The anti-hyperlipidemic activity of *E. prolifera* polysaccharide was evaluated in high-fat diet-induced hyperlipidemic mice. Oral administration of hot water extract of *E. prolifera* polysaccharide (300 mg kg^−1^ body weight) to high fat-fed mice successfully reduced serum total cholesterol (TC), triglyceride (TG), low-density lipoprotein (LDL), and inhibited lipid metabolism in a dose-dependent manner (46). Similarly, Teng et al. (60) reported that dietary supplementation of *E. prolifera* polysaccharide to rats markedly decreased plasma and liver triacylglycerol, total cholesterol (TC), and low-density lipoprotein content.

In another study, a polysaccharide isolated from *E. prolifera* was administered to high-fat diet-induced hyperlipidemic rats. The hypolipidemic activity was assessed in terms of serum and liver TG levels and mRNA expression of hepatic acetyl-CoA carboxylase (ACC). It was observed that the administration of *E. prolifera* polysaccharide to hyperlipidemic rats improved glucose tolerance and insulin resistance, reduced plasma and liver TG and TC levels, and showed the abundance of ACC mRNA expression (61, 62). Moreover, studies have shown that the administration of 200 mg kg^−1^ sulfated *E. prolifera* polysaccharides could attenuate high-fat diet-induced non-alcoholic fatty liver disease in rats (63). It was proposed that the anti-hyperlipidemic effect of *E. prolifera* polysaccharides might be associated with an inhibition of sterol regulatory element-binding protein-1c (SREBP-1c) that resulted in the suppression of biosynthesis of cholesterol (61).

### Antioxidant Activity

Cells undergo an oxidation reaction during the process of energy production. However, this oxidation process may generate excessive reactive oxygen species (ROS, oxygen-derived free radicals), which may adversely affect cells (64, 65). The antioxidant glutathione peroxidase (GSH), superoxide dismutase (SOD), catalase (CAT), and total anti-oxidant (T-AOC) play a decisive role in maintaining body health by removing overproduced ROS. Therefore, it is vital to increase the production of antioxidants in the body to get rid of these free radicals.

Recently, marine algae, including *Enteromorpha prolifera*, have attracted great interest owing to their free radical scavenging activities (66–68). Xu and his colleagues isolated polysaccharides from *E. prolifera* and evaluated their antioxidant activities in terms of their ability to scavenge free radicals. The results showed that *E. prolifera* polysaccharides have antioxidant activity (32). In another study, a water-soluble polysaccharide was extracted from *E. prolifera* using hot water methods and administered to mice fed a high-fat diet to evaluate the antioxidant activities. The results obtained from this study demonstrated that supplementation of *E. prolifera* polysaccharide increased the serum SOD, CAT, GSH-Px activities, and decreased serum malondialdehyde (MDA) content (46). Similarly in chicken, dietary supplementation of *E. prolifera* polysaccharides increased antioxidant levels of T-SOD, GSH-Px, CAT, and, GST and reduced MDA contents in the bursa of Fabricius (69). This may be partially attributed to the activation of the nuclear-related factor 2 (Nrf2) signaling pathway in response to *E. prolifera* polysaccharide supplementation (70).

Research evidence has shown that the antioxidant activities of *E. prolifera* can be influenced by the molecular weight and a sulfate group. In this regard, Li et al. extracted polysaccharides from *E. prolifera* by hot-water method and degraded them into low molecular weight by *P. pabuli*, and then sulfonated with chlorosulfonic acid/pyridine method. The antioxidant activities results showed that the lower molecular weight and sulfated polysaccharides were found to have higher free radical scavenging activities than the higher molecular weight un-sulfated polysaccharides (20).

Furthermore, *E. prolifera* polysaccharide could ameliorate reactive oxygen species accumulation and DNA damage *via* up-regulation of genes, such as protein skinhead-1(SKN-1) and DAF-16, and down-regulation of miR-48, miR-51, and miR-186, suggesting that it has a vigorous antioxidant activity (47). Altogether the above studies suggest that *E. prolifera* polysaccharide has an intense antioxidant activity, and its activity can be enhanced by modifying its chemical composition and molecular weight.

### Antidiabetic Property

The antidiabetic effect of *E. prolifera* has been explored recently in diabetic-induced rats and mice. Intragastric administration of *E. prolifera* polysaccharides to diabetic rats ameliorated glucose metabolism by decreasing the fasting serum blood glucose and insulin levels. This effect might be attributed to promoting antioxidant levels and upregulating the mRNA abundance of glucose and insulin metabolism-related genes, such as glucokinase, insulin receptor, glucose transporter type 4 (GLUT-4), and adiponectin (71). In a recent study, polysaccharide was extracted from *E. prolifera* and enzymatically degraded to produce low molecular weight oligomers. The administration of these oligomers to diabetic mice has shown to relieve diabetic symptoms and reduce pancreatic inflammation and apoptosis, thereby ameliorating streptozotocin-induced diabetes mellitus (72). In addition, a recent study has demonstrated that *E. prolifera* polysaccharide prevents high-fat diet-induced obesity and ameliorated HFD-induced metabolic dysfunction in hamsters (73). Furthermore, administration of *E. prolifera*-chromium (III) complex to mice fed a high-fat and high-sucrose diet could improve glucose tolerance and reduce serum insulin levels *via* activation of the *IR/IRS-2/PI3K/PKB/GSK-3*β signaling pathway, suggesting that it could be a potential therapeutic agent against type 2 diabetes (74).

### Gut Microbiota Modulation

It is well established that diet and nutritional factors have a direct effect on the microbial colonization of the gut (75–77). A growing body of evidence suggests that gut microbiota is involved in the digestion and utilization of fibers such as polysaccharides, which otherwise cannot be utilized by the host. These microbes produce short-chain fatty acids as an end product from the diet, which has an important role in energy metabolism (78). Apart from this, gut microbiota play a decisive role in maintaining the homeostasis and health of the host *via* direct involvement in gut structure and morphology, regulation of immune responses, and protection from luminal pathogens. Therefore, any factor that leads to microbiota dysbiosis may affect the health status and immune response of the host. Recently, Kong et al. (79) extracted sulfated polysaccharides from *E. prolifera* with hot water method and fermented them *in vitro* for 48 h by human fecal cultures. Those authors observed that human fecal cultured with *E. prolifera* polysaccharides produced more short-chain fatty acids (SCFAs), including butyrate, acetate, and lactic acid, and increased the production of beneficial intestinal *Lactobacillus* bacteria (79). Thus, it was concluded that *E. prolifera* polysaccharides could exert a prebiotic effect in humans by promoting SCFAs production and regulating the intestinal microbiota.

Similarly, the modulatory effects of *E. prolifera* polysaccharide on gut microbiota were also investigated in mice. For this purpose, water extracted *E. prolifera* polysaccharides were administered to mice for 2 weeks, and then the fecal pellets were analyzed using the 16S-rRNA sequencing approach. The results showed that *E. prolifera* polysaccharide could modulate intestinal microbiota (80). Furthermore, a metagenomic sequencing analysis study in the intestinal microbiota of rabbit fish *S. oramin* showed that *E. prolifera* diet could increase the abundance of *Bacteroidetes* bacteria, suggesting that this bacterium is responsible for the digestion of *E. prolifera* (81). All results indicated that *E. prolifera* polysaccharides could modulate gut microbiota and enhance SCFAs production, thereby regulating the health and homeostasis of the host. Further research is required to elucidate the influence of these polysaccharides on the immune-microbiome interactions at a cellular and molecular level.

### Miscellaneous Activities

Apart from the above-mentioned health benefits, *E. prolifera* polysaccharides were found to have other biological properties, such as removal of environmental pollutants, anticoagulant, antibacterial, anticancer, anti-tumor drug delivery, iron-chelating, moisture retention, gelling property, and growth-promoting effect.

#### Removal of Environmental Pollutant

*E. prolifera* can be used for the removal of environmental pollutants. In this regard, Zhao et al. (37) used *E. prolifera* with polyaluminum chloride as a coagulant aid to remove silver nanoparticles-humic acid contaminant. The author reported that when *E. prolifera* and polyaluminum chloride were used at doses of 0.3 mg L^−^1 and 2.0 mg L^−^1, respectively, the silver nanoparticles were completely removed through the coagulation-ultrafiltration process and the membrane flux was improved. Zhao et al. (37) investigated the coagulant effect of *E. prolifera* polysaccharide in terms of organics removal, floc properties, and membrane fouling degree and found that it has higher organics removal and lower membrane fouling properties, indicating that it is a potential coagulant aid.

#### Anticoagulant Activity

Cui et al. (55) evaluated the anticoagulant activity of sulfated *E. prolifera* polysaccharides in terms of activated partial thromboplastin time (APTT), thrombin time (TT), and prothrombin time (PT) *in vitro* and was found these polysaccharides could be a potential anticoagulant agent.

#### Antimicrobial Activity

*E. prolifera* polysaccharide has been reported to have an antibacterial effect. For example, Shao et al. (22) observed that hydroxamate degraded polysaccharides exhibit a higher inhibitory effect against the gram-positive (*Bacillus subtilis* and *Staphylococcus aureus*) and gram-negative (*Salmonella, pseudomonas aeruginosa*, and *Escherichia coli*) bacteria (22). Likewise, in an experimental condition, the *E. prolifera* polysaccharide-selenide complex showed an inhibitory effect against *E. coli* and *S. aureus* (82).

#### Anticancer Activity and Drug Delivery

Recently, sulfated polysaccharides were extracted from *E. prolifera* using the hot water method, and their anti-cancer activity was evaluated *in vitro* and BALB/c-nu mice. The results demonstrated that *E. prolifera* polysaccharide could inhibit human lung cancer cell proliferation *in vitro*. Besides, the authors reported that administration of 100 mg/kg sulfated *E. prolifera* polysaccharide to mice inhibited tumor by 59 %, suggesting that this polysaccharide might be a good candidate for the treatment of lung cancer (83). In addition, *E. prolifera* polysaccharide has been reported as a promising candidate for anti-tumor drug delivery (84).

#### Thicker, Iron Chelating, and Moisture Absorption Properties

It has been reported that 16 g/L *E. prolifera* polysaccharide could form a gel and because of this gelling properties, it can be used as a thickening agent in the food industry (14). In addition, studies have shown that sulfated polysaccharides from *E. prolifera* were found to have an iron-chelating capacity (25). Furthermore, *P. pabuli* enzymatic hydrolysis and chlorosulfonic acid sulfate group modification of *E. prolifera* polysaccharides resulted in moisture absorption/water retention capabilities (20).

#### Effect on Skin

*E. prolifera* polysaccharide could alleviate hydrogen peroxide-induced injuries on human skin fibroblasts, suggesting that it may play roles in the cosmetics industry (85).

#### Effect of *E. prolifera* Polysaccharides on Other Animal Species

The growth promoter effect of *E. prolifera* polysaccharides in crucian carp was evaluated in terms of body weight gain, feed conversion ratio, and body crude protein content. The results revealed that dietary supplementation of *E. prolifera* polysaccharide (40 g kg^−1^ diet) was found to improve growth performance and enhance digestive enzyme activity in crucian carp (86). Similarly, studies in broiler chicken showed that dietary supplementation of 0.5–1% *E. prolifera* polysaccharide could improve growth performance and immune function (87).

## Conclusion and Perspectives

*Enteromorpha prolifera* is a green alga with worldwide distribution, which has been used as a medicine and food. Recently, a water-soluble sulfated polysaccharide isolated from *E. prolifera* gains growing interest by scientists due to its proven physiological and biological activities, including gut microbiota modulation, immunomodulation, anti-oxidant, anti-bacterial, anti-hyperlipidemia, and anti-diabetic properties. *E. prolifera* polysaccharides can be isolated by various techniques, of which the microwave-assisted extraction method has been reported to be efficient. Structural modification of *E. prolifera* polysaccharides results in improvements in biological activities. Therefore, further studies should be focused on the architecture-activity relationship to enhance the function and promote its utilization. In addition, the effect of *E. prolifera* polysaccharides on the antimicrobial activity should be examined using different assays such as zone of inhibition, MIC-assay, biofilm formation inhibition assay, and quorum sensing inhibition assay to promote the application of these polysaccharides as an antibiotic alternative. Although numerous studies have reported the pharmacological activity of *E. prolifera* polysaccharide, its mechanism of action has not been fully studied. Thus, further insights into the molecular mechanism by which *E. prolifera* polysaccharides modulate gut microbiota are needed for better use of this polysaccharide as a functional food and therapeutic purpose.

## Author Contributions

TW, KN, and HW carried out the literature study and drafted the manuscript. CX and WX critically evaluated the manuscript. All authors checked, revised, and approved the final manuscript.

## Funding

We would like to acknowledge NSFC (31902196), Science and Technology Projects of Hunan Province (2019RS3020), the earmarked fund for China Agriculture Research System (CARS-35), China Postdoctoral Science Foundation-funded project (2021M693383, 2019M662273), and Taisha industry leading talent blue talent project for their financial support.

## Conflict of Interest

HW was employed by Qingdao Seawin Biotech Group Co., Ltd., Qingdao, China. The remaining authors declare that the research was conducted in the absence of any commercial or financial relationships that could be construed as a potential conflict of interest.

## Publisher's Note

All claims expressed in this article are solely those of the authors and do not necessarily represent those of their affiliated organizations, or those of the publisher, the editors and the reviewers. Any product that may be evaluated in this article, or claim that may be made by its manufacturer, is not guaranteed or endorsed by the publisher.
